# Temporal analysis and contextual factors associated with HIV/AIDS in Brazil from 2000 to 2019

**DOI:** 10.11606/s1518-8787.2023057005508

**Published:** 2023-11-17

**Authors:** Denise Eliziana de Souza, Cleber Nascimento do Carmo, James R. Welch

**Affiliations:** I Fundação Oswaldo Cruz Escola Nacional de Saúde Pública Sérgio Arouca Rio de Janeiro RJ Brazil Fundação Oswaldo Cruz. Escola Nacional de Saúde Pública Sérgio Arouca. Graduate Program in Public Health Epidemiology. Rio de Janeiro, RJ, Brazil

**Keywords:** HIV Infection, Acquired Immunodeficiency Syndrome, Time Series Studies, Social Determinants of Health

## Abstract

**OBJECTIVE:**

To describe the profile of the HIV/AIDS epidemic in Brazil and its Federation Units by gender, identify its associated contextual factors, and track changes in its epidemiological pattern from 2000 to 2019.

**METHODS:**

This is an ecological study with epidemiological data from DATASUS and population data from the Brazilian Institute of Geography and Statistics. Time-series analyses of incidence rates by gender and trends were performed by joinpoint regressions, obtaining the average annual percent change (AAPC). Then, all genders were analyzed regarding the association between AAPC and the following contextual indicators: Municipal Human Development Index (HDI-M), Gini Index, Social Vulnerability Index, illiteracy rates, proportion of late diagnosis, and proportion of test distribution.

**RESULTS:**

Incidence rates in men showed a linear decreasing trend (AAPC = −0.6; 95%CI −1.1 to 0.0). Rates in women increased from 2000 to 2009 and decreased from 2010 to 2019, tending upward throughout the period (AAPC = 1.4; 95%CI 0.8 to 1.9). Analyses by gender ratio showed a downward trend (AAPC = −1.8; 95%CI −2.3 to −1.3), indicating a reduction in the rates in men when compared to women. Indicators and the AAPC showed an inverse association for all genders, in which the HDI-M was the variable with the most pronounced association, showing that higher human development indices are associated with lower variations in HIV/AIDS rates.

**CONCLUSION:**

Case distribution differ across genders, with an upward incidence trend in women and a possible association with gender-related vulnerabilities. It is important to think about public policies that consider these dimensions.

## INTRODUCTION

The global HIV/AIDS epidemic has cases worldwide^1^. It is estimated that 38.4 million people lived with HIV/AIDS worldwide in 2021^2^. From the beginning of the epidemic^3^ to June 2022, Brazil reported 1,088,536 cases. In 2021, 54% of people living with HIV worldwide were women and girls, with heterosexual intercourse configuring the main category of exposure in women, corresponding to 86.6% of cases and especially reaching the female population in reproductive age, 45.6% of which including the age group from 15 to 34 years, with implications regarding the possibility of vertical transmission of the infection^2,3^.

Social inequalities and changes in the predominance of transmission routes over time contribute to the diverse epidemiological profiles of the epidemic^1^. Initially restricted to men who had sex with men, those who injected drugs, and people living with hemophilia, the contours of the epidemic have changed, reaching women more widely. An upward trend in cases of HIV infection in women has been observed since the early 1990s, markedly characterized by heterosexual transmission and associated with factors related to greater female vulnerability, especially due to the ways with which men and women relate to each other in society and the power dynamics permeating these relationships^4,5^.

This study hypothesized that the distributions of HIV and AIDS cases in Brazil and in its female population differ from each other and that, when compared to the male population over the years and per Federation Unit (FU), it has followed social determinants measured by contextual indicators.

This study aims to describe the profile of the HIV/AIDS epidemic in Brazil according to gender and FU to evaluate its associated contextual factors and monitor changes in its epidemiological pattern from 2000 to 2019 in individuals aged 13 years or above. This research estimated HIV/AIDS incidence rates and their trends in Brazilian states by gender and ratio, associations between contextual indicators in Fus, and the average annual percentage change of HIV/AIDS.

This study evaluates the last two decades of the pandemic to help to contextualize HIV/AIDS incidence rates regarding gender-related vulnerabilities and find conditions for which it would be possible to propose health practices and public policies that minimize inequalities.

## METHODS

### Study Design and Population

The incidence rates of HIV/AIDS in men and women aged 13 years or above were considered in this ecological study^6^. The cut-off point for the age group was based on the definition criteria for reporting the disease^7^.

The study consisted of two stages: rate time series analysis^8^ and evaluation of possible associations between the average rate percentage change and contextual factors.

### Data Sources

Information on cases was obtained from the open-access Brazilian Notifiable Diseases Information System database^9^. Variables were chosen according to notification period in each state and stratified by gender. Age groups from 13 years and above and the gender variable (stratified into man and women categories) were used. The variables self-reported race/color, schooling, and exposure hierarchy were used to characterize the sample.

Population data were obtained from projections and estimates from the demographic census of the Brazilian Institute of Geography and Statistics^10^. Contextual variables were obtained from data from the United Nations Development Programme^11^, the Institute for Applied Economic Research ^12^, and the Brazilian Ministry of Health^13^.

The Municipal Human Development Index^11^ (HDI-M) has dimensionless measures from 0 to 1 that classify values from very high to very low, with values closer to 1 indicating greater human development. The Gini^14^ and Social Vulnerability Indices^12^ (SVI) also contain measures from 0 to 1, in which higher values indicate more unfavorable conditions. The proportion of test distribution^13^ refers to the ratio between the number of rapid HIV diagnostic tests distributed in each state and the total number of tests distributed in the country. Illiteracy rate^14^ refers to the proportion of individuals aged 15 years or above among all those in the same age group who reported an inability to read or write. The proportion of late diagnoses^15^ refers to the proportion of individuals with results below 200 cells/mL among all HIV-infected individuals who underwent the first CD4+ T lymphocyte test and who were yet to begin treatment. The contextual indicators in this study refer to each Brazilian FU. The most recent available values were obtained for the studied period.

### Temporal Analysis of HIV/AIDS Incidence Rates in Brazil

The time series analysis considered the years of the study period (January 1, 2000, to December 31, 2019) and the annual incidence rates of HIV/AIDS as independent and dependent variables, respectively. The number of reported cases of HIV/AIDS in each state and the population living in each state in the period by gender were considered to calculate incidence rates. Incidence rates were standardized by the direct method^16^. The ratio between the incidence rates for both genders was also analyzed by the ratios between the rates for men and those for women.

Polynomial regression models were used to relate incidence rates (Y) with time in years (X)


$$\text{Y}={\text{β}}_{0}+{\text{β}}_{1}\text{X}+{\text{β}}_{2}{\text{X}}^{2}+\in$$

in which the statistical significance of β_2_ corresponds to the quadratic term of the regression and indicates a better representation of the data by nonlinear relationships. For these analyses, the variable time (in years) was centralized to avoid data autocorrelation.

A joinpoint regression^17^ was used to describe temporal evolution by inflection points indicating trend change. A model — given the observations (x_1_, y_1_),…,(x_n_, y_n_, ), in which x_1_,≤…≤ x_n_ and with τ_k_ unknown junction points — can be written by


$$\text{E}\left[\text{Y}|\text{X}\right]={\text{β}}_{0}+{\text{β}}_{1}\text{X}+{\text{δ}}_{1}{\left(\text{X}-{\text{τ}}_{1}\right)}^{+}\text{+}\cdots {\text{+δ}}_{\text{k}}{\left(\text{X}-{\text{τ}}_{\text{k}}\right)}^{+}$$

The inclusion of junction points was tested by the Monte Carlo permutation, which uses a sequence of tests from a sample of possible permutations with the Bonferroni correction^18^.

The annual (APC) and average annual percent changes (AAPC) for the whole period were obtained by this method. APC corresponds to a linear change in percentage rates, estimated on a logarithmic scale and considering that x_1_,…,x_n_ represents the years of the studied period and y_1_,…,y_n_, the logarithm of the observed rates, in which log (T_y_) refers to the logarithm of the rate in year y. The percentage change in rates from year y to year y + 1 corresponds to


$$\left[\frac{{\text{T}}_{\text{y}+1}+\text{Ty}}{\text{Ty}}\right]\times 100$$

Thus, the APC is the percentage obtained by exponentially increasing the difference between the regressions of each period and its preceding interval. The AAPC, in turn, corresponds to a summary measure of the trend over a fixed time interval that is calculated by the weighted average of APCs, in which weights equal the length of the interval.


$$\text{AAPC}=\left\{\exp\left(\frac{\sum {\text{w}}_{\text{i}}{\text{b}}_{\text{i}}}{\sum {\text{w}}_{\text{i}}}\right)-1\right\}\times 100$$

In which b_i_ refers to the slope coefficient for the ith segment and w_i_ the length of each segment in the interval of years.

### Association of AAPC with Contextual Indicators

The HIV and AIDS AAPC obtained in the temporal analysis was considered the dependent variable (Y) in the evaluation of possible associations between average percentage change in HIV/AIDS rates and contextual indicators. Contextual indicators were used as independent variables (X) in this study. Linear regression models were estimated for univariate and multiple analyses.

A 5%statistical significance was considered in the analyses, which were performed on R^19^, version 4.1.3, and Joinpoint Regression Program^17^, version 4.9.1.0.

### Ethical Considerations

The use of secondary open-access data dispensed the submission of this study for consideration by the Research Ethics Committee^20^.

## RESULTS

### Sociodemographic and epidemiological variables

A total of 498,543 cases of HIV/AIDS were diagnosed in men (64.01%) and 280,322 cases in women (35.99%) aged 13 years or above from 2000 to 2019. Men showed the highest rates: the lowest corresponds to 28.70/100,000 in 2006 and the highest, to 33.42/100,000 in 2000. The lowest rate in women totaled 7.84/100,000 in 2000 and the highest, 12.96/100,000 in 2009.

The population living with the disease (Table 1) shows a significant proportion of cases in young people and adults aged from 20 to 49 years (83.22 and 80.97% for men and women, respectively), and in individuals with incomplete primary education show a higher proportion, especially women (60.30%). Black and mixed-race individuals show a higher proportion (46.20% for men and 30.41% for women). Sexual transmission occurred most often, especially in heterosexuals of all genders, more so in women (59.93%). The “unknown” category showed important proportions for race/color (37.55% for men and 40.43% for women) and exposure hierarchy (42.57% for men and 36.72% for women).

**Table 1:** Sociodemographic characteristics of HIV/AIDS cases by gender in Brazil from 2000 to 2019.

Characteristics		Male		Female	
		n	%	n	%
Age group					
	13–19	8,864	1.78	8,060	2.87
	20–29	120,643	24.2	66,575	23.75
	30–39	174,191	34.94	93,709	33.43
	40–49	120,076	24.08	66,701	23.79
	50–59	53,761	10.78	32,267	11.51
	≥ 60	21,008	4.21	13,010	4.64
Schooling					
	Illiterate	7,901	2.9	6,318	4.84
	Incomplete primary education	113,393	41.6	78,774	60.3
	Complete primary education	23,583	8.65	14,377	11.01
	Incomplete secondary education	37,082	13.6	19,834	15.18
	Complete secondary education	46,115	16.92	1,919	1.47
	Incomplete higher education	12,469	4.57	2,348	1.8
	Complete Higher Education	32,056	11.76	7,056	5.4
Race/color					
	White	156,273	31.34	79,737	28.44
	Black	32,386	6.5	21,574	7.7
	Yellow	1,773	0.35	929	0.33
	Mixed-race	120,052	24.08	64,225	22.91
	Indigenous	871	0.17	518	0.18
	Ignored	187,188	37.55	113,339	40.43
Exposure Hierarchy					
	Homosexual	87,481	17.55	1,841	0.66
	Bisexual	29,718	5.96	811	0.29
	Heterosexual	144,145	28.91	167,997	59.93
	UID	23,221	4.66	5,534	1.97
	Hemophiliac	199	0.04	0	0
	Transfusion	130	0.03	106	0.04
	Accident with biological materials	12	0.002	8	0.002
	Vertical transmission	1,408	0.28	1,111	0.4
	Ignored	212,229	42.57	102,914	36.72

UID: users of injectable drugs.

### Time Series Evolution

Trend analysis by FU (Table 2) shows a statistically significant upward trend (AAPC > 0) in incidence rates for Northern and Northeastern FUs in all genders and greater declining rate trends (AAPC < 0) in Southern and Southeastern FUs.

**Table 2:** Trend analysis of HIV/AIDS rates by gender in Brazilian federative units from 2000 to 2019.

FU	Gender	Beta1	Beta2	R²	AAPC (%)
Acre (AC)	Men	0.51	-	0.58	**4.3**
	Women	0.02	−0.04	0.27	0.7
Alagoas (AL)	Men	0.99	0.03	0.92	**5.4**
	Women	0.36	−0.03	0.78	**4.6**
Amapá (AP)	Men	1.58	-	0.82	**5.9**
	Women	0.87	−0.06	0.73	**6.4**
Amazonas (AM)	Men	2.26	−0.15	0.85	**6.1**
	Women	0.55	−0.11	0.75	4.3
Bahia (BA)	Men	0.49	−0.03	0.9	**2.8**
	Women	0.15	−0.04	0.8	**1.7**
Ceará (CE)	Men	0.75	−0.02	0.93	**3.5**
	Women	0.08	−0.04	0.75	1.6
Federal District (DF)	Men	−0.31	−0.09	0.29	−0.7
	Women	−0.65	-	0.75	**−4.2**
Espírito Santo (ES)	Men	0.23	−0.07	0.48	0.5
	Women	−0.49	−0.07	0.78	−2.2
Goiás (GO)	Men	0.37	-	0.68	1.6
	Women	−0.25	−0.02	0.74	−2.2
Maranhão (MA)	Men	1.39	−0.05	0.94	**6.3**
	Women	0.68	−0.07	0.93	**5.7**
Mato Grosso (MT)	Men	0.73	-	0.75	**2.6**
	Women	−0.11	−0.08	0.5	1.4
Mato Grosso do Sul (MS)	Men	0.84	-	0.7	**2.9**
	Women	0.09	−0.07	0.42	0.1
Minas Gerais (MG)	Men	−0.05	−0.02	0.25	−0.2
	Women	−0.39	−0.04	0.85	**−2.8**
Pará (PA)	Men	1.83	-	0.95	**8.5**
	Women	0.86	−0.06	0.93	**8.6**
Paraíba (PB)	Men	0.72	-	0.91	**4.1**
	Women	0.15	−0.04	0.73	**2.9**
Paraná (PR)	Men	-	-	0.05	0.3
	Women	−0.37	−0.05	0.67	−1.7
Pernambuco (PE)	Men	0.79	−0.05	0.91	**3.7**
	Women	0.21	−0.09	0.88	**2.4**
Piauí (PI)	Men	0.71	−0.05	0.8	**3.9**
	Women	0.27	−0.05	0.83	**4**
Rio de Janeiro (RJ)	Men	−0.61	−0.06	0.76	**−1.6**
	Women	−0.66	−0.08	0.87	**−2.1**
Rio Grande do Norte (RN)	Men	1.29	-	0.9	**7.3**
	Women	0.34	-	0.67	**4.5**
Rio Grande do Sul (RS)	Men	−0.51	−0.13	0.71	**−1.6**
	Women	−0.51	−0.2	0.86	−0.9
Rondônia (RO)	Men	1.02	−0.06	0.76	4
	Women	0.01	−0.11	0.82	−0.1

AAPC: average annual percent change.

Note: Values in bold indicate a statistical significance at a level of 5%.

Ratio analysis by gender between incidence rates indicates a quadratic trend and the analysis by inflection points, a downward trend in the ratio between rates by gender (AAPC = −1.8; 95%CI −2.3 to −1.3). Gender ratios decreased from 2000 to 2008, slightly increasing from 2009 to 2019. The ratio of incidence rates between genders shows a statistically significant downward trend in 17 FUs, indicating an increase in the proportions of incidence rates in women in relation to men.

**Figure 1:**
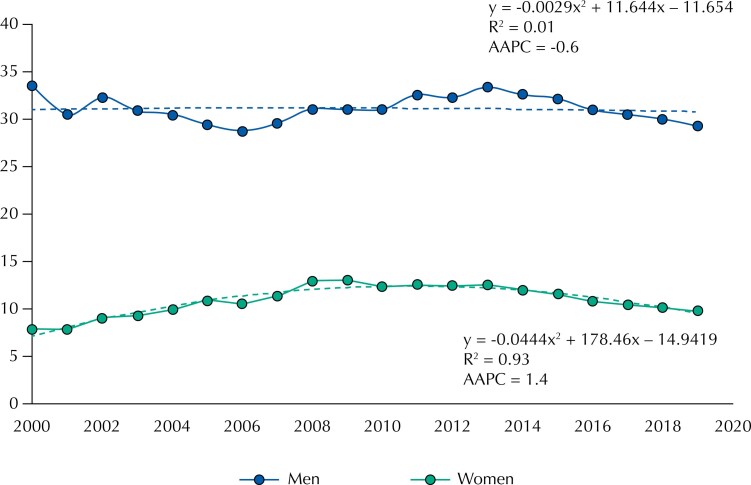
Trend of HIV/AIDS rates (per 100,000) by gender in Brazil from 2000 to 2019.

Note: Values in bold indicate a statistical significance at a level of 5%.

In Brazil, polynomial regression analyses (Figure 1) indicate a linear trend of decline in incidence rates in men and the analysis by inflection points showed a general downward trend in rates (AAPC = −0.6; 95%CI −1.1 to 0.0). The model for women shows a quadratic trend with an increase in incidence rates from 2000 to 2009 and a decline from 2010 to 2019. Segmented regression analysis shows a general upward trend in rates for the whole studied period (AAPC = 1.4; 95%CI 0.8 to 1.9).

### Association of AAPC with Contextual Indicators

Among the statistically significant univariate models (Table 3), HDI-M showed the highest coefficient values for men and women (61.31 and 71.9, respectively). The univariate models for illiteracy rates, late diagnosis proportion, and SVI are directly associated with AAPC, whereas HDI-M and proportion of test distribution are inversely associated. The Gini Index showed no statistical significance.

**Table 3:** Results of univariate regression analyses of the average annual percentage change (AAPC) of HIV/AIDS rates in men and women in Brazil from 2000 to 2019.

Characteristic		Beta1	R²	p-value
Men				
	Illiteracy rate	0.39	0.39	**< 0.001**
	HDI-M	−61.31	0.69	**< 0.001**
	Gini Index	24.74	0.12	0.07
	Proportion of late diagnosis	0.37	0.24	**< 0.01**
	Social Vulnerability Index	29.06	0.29	**< 0.01**
	Test Distribution Ratio	−0.63	0.36	**< 0.001**
Women				
	Illiteracy rate	0.46	0.4	**< 0.001**
	HDI-M	−71.9	0.71	**< 0.001**
	Gini Index	30.79	0.14	0.05
	Proportion of late diagnosis	0.45	0.26	**< 0.01**
	Social Vulnerability Index	33.76	0.29	**< 0.01**
	Test Distribution Ratio	−0.57	0.22	**0.01**

HDI-M: Municipal Human Development Index.

Note: Values in bold indicate a statistical significance at a level of 5%.

The multiple model for men (Table 4) shows a statistical significance for illiteracy rates, HDI-M, and test distribution proportion. For women, only the HDI-M maintains statistical significance when combined with the other independent variables.

**Table 4:** Results of multiple regression analysis of the average annual percentage change (AAPC) of HIV/AIDS rates in men and women in Brazil from 2000 to 2019.

Characteristic (men)		Coefficients	p-value
Men			
	Illiteracy rate	−0.26	**0.03**
	HDI-M	−64.98	**< 0.001**
	Gini Index	10.29	0.29
	Proportion of late diagnosis	0.1	0.18
	Social Vulnerability Index	3.67	0.69
	Test Distribution Ratio	−0.39	**< 0.001**
Women			
	Illiteracy rate	−0.3	0.05
	HDI-M	−83.72	**< 0.001**
	Gini Index	15.67	0.22
	Proportion of late diagnosis	0.13	0.19
	Social Vulnerability Index	−0.48	0.97
	Test Distribution Ratio	−0.25	0.06

HDI-M: Municipal Human Development Index.

Note: Values in bold indicate a statistical significance at a level of 5%.

The analysis of the estimated coefficients for the multiple model for men indicates that the average annual percentage of HIV/AIDS rates decreases by 26% for each percentage increase in illiteracy rates. AAPC decreases by about 65% for every HDI-M increase (on a scale of 0 to 1). The average percentage change in the rates of the disease decreases by 39% for each percentage increase in test distribution proportion. Regarding women, AAPC decreases by about 83% for each increase from 0 to 1 in the HDI-M if conditioned to the other predictors in the complete model.

## DISCUSSION

Variations in the ratios of incidence rates by gender show that the proportion of rates for men and women shows a general downward trend during the studied period. However, trends differ across states. Aspects such as social inequalities; vulnerabilities; trend changes; and the extent of the disease in different population segments, regions, and communities have been associated with differences in gender rates and locations^3,4^. Studies on rate trends according to gender conducted in different Brazilian states show variations in the ratios of these variables, although the proportion of cases among men always exceeds that of women^21^.

Studies on epidemic trends have found that rate changes coincide with the implementation of health policies and practices (i.e., treatment as prevention) aiming to interrupt the chain of transmission, implement prenatal testing (which has proven to be an important diagnostic opportunity for women of reproductive age), and establish compulsory notification for cases of HIV infection without an immunodeficiency diagnosis. Epidemiological trends are consistent with the benefits of early detection policies and diagnosed individuals’ access to therapeutic follow-up^22,23^.

This study found that HIV/AIDS incidence rates show higher magnitudes and greater upward trends in average rate variations in Northern and Northeastern states, whereas Southern and Southeastern states show lower rate magnitudes and declining trends. The epidemic, which began in São Paulo State, has geographically spread to Northern and Northeastern states since the 1990s, associated with migratory flows in border regions. This dispersion pattern involving complex social inequality dynamics contributed to the contours of the epidemic. Rate trends in these regions have become more pronounced^24^.

The analysis of contextual indicators highlights different dimensions of inequalities across FUs. Results point to greater socioeconomic vulnerability in the Brazilian North and Northeast than in its South and Southeast. HDI-M is the indicator with the greatest association with annual rate percentage variation, showing statistical significance for both strata in all analyses and a more pronounced association for women. It is worth specifying that the large values of the coefficients estimated for this indicator in the multiple model can be derived from the HDI-M scale, which ranges from 0 (minimum) to 1 (maximum). Thus, the increase in one unit in the HDI-M variable is equivalent to a 100% increase in the scale, from the worst to the best condition. A study on temporal and spatial trends and social determinants of HIV/AIDS also found a positive association for HDI-M^25^. This study found a statistically significant proportion of late diagnosis and SVI in its univariate analyses, which disappeared when combined with other contextual indicators. Another study showed a high prevalence of late diagnosis among men, pointing to persistent barriers in their access to health care (including HIV testing)^26^. Another study used SVI in its analysis of factors associated with tuberculosis, showing a greater association with rates in more vulnerable municipalities^27^. Our results show a statistically significant proportion of test distribution and illiteracy rates in our univariate analyses for both strata, which only remained significant in the multiple analyses only for men. A study on the distribution of tests points to a significant increase since its implementation and better provision to states throughout the country^28^. Lower schooling rates configure a factor that impairs several health conditions and has been associated with higher number of HIV/AIDS diagnoses in Brazil^29^. Although the Gini Index showed no statistically significant association in our analyses, another study found an association between indicators of better socioeconomic conditions and HIV infection, suggesting inequality in access to the diagnosis of infection^30^.

This study found a predominance of cases and incidence rates, with higher magnitudes in men than in women and transmission primarily referring to sexual intercourse. These findings resemble official data from the Ministry of Health^3^ and from studies on epidemiological profiles in Brazil^31^. It is observed that case proportion remains higher in men over time (although the heterosexual route has become more pronounced), corroborating the case distribution profiles in this study. Recent studies point to an increase in incidence in younger age groups and those with low schooling, which may indicate behavioral changes related to preventive practices, differences in access to diagnosis, unequal availability of health services for the national population, and the role of race and ethnic identity as social determinants^32^. Thus, finding the more vulnerable population segments and implementing targeted health actions can promote social equality and health equity and improve the profile of the epidemic in Brazil.

The limitations of this study include its use of a secondary quantitative database as it prohibits a qualitative investigation of the plurality of women's lives in Brazil, such as gender perceptions, sexual practices, and vulnerability contexts. Moreover, notification forms showed gaps regarding variables such as race/color and exposure hierarchy. Although time-series studies can observe the behavior of rates over time, analyses are subject to varying diagnostic criteria and the availability of robust and consistent data. However, the possibility of situating trends in their historical and social contexts can better explain the changing points. It should also be noted that, as this is an ecological study, it is impossible to make inferences at the individual level regarding results, but this restriction fails to compromise findings since the proposed objectives targeted the population level.

## CONCLUSION

The epidemic has different contours according to gender. Despite the predominance of cases in men, the ratio between incidence rates by gender differs by region and period. HIV/AIDS cases are unlikely distributed in men and women, tending toward increasing incidence rates in women and containing a greater magnitude of the effect of the human development indicator in Brazil, which may point to an association between the increase in rates in women and gender-related vulnerabilities. It is important to think about public policies that consider these dimensions.
