# Supplementary material for: Temporal analysis and contextual factors associated with HIV/AIDS in Brazil from 2000 to 2019
Source: Rev Saude Publica. 2023 Nov 17;57:91. doi: 10.11606/s1518-8787.2023057005508 (PMC10681528; doi:10.11606/s1518-8787.2023057005508)
Supplement: Translation

# Translation: Análise temporal e fatores contextuais associados ao HIV/aids no Brasil entre 2000 e 2019

**Authors:** Denise Eliziana de Souza, Cleber Nascimento do Carmo, James R. Welch

**Affiliations:** I Rio de Janeiro RJ Brasil Fundação Oswaldo Cruz. Escola Nacional de Saúde Pública Sérgio Arouca. Programa de Pós-graduação em Epidemiologia em Saúde Pública. Rio de Janeiro, RJ, Brasil

**Keywords:** Infecção por HIV, Síndrome de Imunodeficiência Adquirida, Estudos de Séries Temporais, Determinantes Sociais da Saúde

## Abstract

**OBJETIVO:**

Descrever o perfil da epidemia de HIV/aids no Brasil e nas unidades da federação de acordo com o sexo, identificar os fatores contextuais associados e acompanhar mudanças no padrão epidemiológico entre 2000 e 2019.

**MÉTODOS:**

Estudo ecológico utilizando dados epidemiológicos do Datasus, e populacionais do Instituto Brasileiro de Geografia e Estatística (IBGE). Inicialmente foram realizadas análises de séries temporais das taxas de incidência por sexo e de tendências por regressões *joinpoint*, com obtenção da média da variação percentual das taxas (*average annual percent change –* AAPC). Posteriormente procedeu-se a uma análise, para ambos os sexos, da associação da AAPC com os indicadores contextuais Índice de Desenvolvimento Humano Municipal (IDH-M), Índice de Gini, Índice de Vulnerabilidade Social, taxa de analfabetismo, proporção de diagnóstico tardio e proporção de distribuição de testes.

**RESULTADOS:**

Observa-se tendência linear de redução nas taxas de incidência para o sexo masculino (AAPC = −0,6; IC95% −1,1 a 0,0). Para o sexo feminino, houve aumento nas taxas entre 2000 e 2009 e declínio entre 2010 e 2019, com uma tendência de incremento no período completo (AAPC = 1,4; IC95% 0,8 a 1,9). As análises por razão entre os sexos indicaram tendência de declínio (AAPC = −1,8; IC95% −2,3 a −1,3), apontando redução nas taxas para o sexo masculino em relação ao sexo feminino. Verificou-se associação inversa dos indicadores com a AAPC para ambos os sexos, sendo IDH-M a variável com associação mais pronunciada, evidenciando que maiores índices de desenvolvimento humano estão associados a menores variações nas taxas de HIV/aids.

**CONCLUSÃO:**

Os casos se distribuem de formas distintas entre os sexos, com tendência de incremento na incidência em mulheres e possível associação com vulnerabilidades relacionadas ao gênero, sendo importante pensar em políticas públicas que considerem essas dimensões.

## INTRODUÇÃO

A epidemia de HIV/aids é de escala global, com casos identificados nas diferentes regiões do mundo^1^. É estimado que em 2021 havia 38,4 milhões de pessoas vivendo com HIV/aids no mundo^2^. No Brasil, do início da epidemia^3^ até junho de 2022 haviam sido notificados 1.088.536 casos. Em 2021, 54% de todas as pessoas vivendo com HIV no mundo eram mulheres e meninas, sendo a via heterossexual a principal categoria de exposição entre mulheres, correspondendo a 86,6% dos casos e alcançando em grande parte a população feminina em idade reprodutiva, 45,6% na faixa etária de 15 a 34 anos, com implicações quanto à possibilidade de transmissão vertical da infecção^2,3^.

As desigualdades sociais e as mudanças na predominância das vias de transmissão ao longo do tempo contribuem para que a epidemia apresente perfis epidemiológicos diversos^1^. Inicialmente restrita a homens que fazem sexo com homens, pessoas que fazem uso de drogas injetáveis e com hemofilia, os contornos da epidemia mudaram, tendo maior alcance entre as mulheres. Uma tendência de aumento de casos de infecção por HIV em indivíduos do sexo feminino já era observada desde o início da década de 1990, caracterizada, de forma marcante, pela via de transmissão heterossexual e associada a fatores relacionados a uma maior vulnerabilidade feminina devido principalmente às formas como homens e mulheres se relacionam na sociedade e às dinâmicas de poder que perpassam essas relações^4,5^.

As hipóteses testadas neste estudo foram de que a distribuição dos casos de HIV e aids no Brasil e na população feminina têm ocorrido de maneiras diferentes entre si e quando comparadas à população masculina, ao longo dos anos e em cada uma das Unidades da Federação (UF), e que tem sido acompanhada por determinantes sociais mensurados por indicadores contextuais.

O objetivo deste estudo é descrever o perfil da epidemia de HIV/aids no Brasil de acordo com o sexo e em cada uma das UF, avaliar fatores contextuais associados e acompanhar mudanças no padrão epidemiológico no período de 2000 a 2019 em indivíduos com 13 anos ou mais. Foram estimadas taxas de incidência de HIV/aids e suas tendências nas UF por sexo e por razão destas taxas. Também foram analisadas associações entre indicadores contextuais nas UF com a média da variação do percentual anual de HIV/aids.

Este estudo, referente às últimas duas décadas da pandemia, busca contribuir com a contextualização das taxas de incidência de HIV/aids em termos de situações de vulnerabilidades relacionadas aos sexos, possibilitando, então, identificar condições em que seja possível propor práticas em saúde e políticas públicas que minimizem desigualdades.

## MÉTODOS

### Delineamento e População do Estudo

Este estudo ecológico^6^ considerou as taxas de incidência de HIV/aids em indivíduos dos sexos masculino e feminino, com 13 anos ou mais. O ponto de corte para a faixa etária foi baseado nos critérios de definição para notificação do agravo^7^.

O estudo foi composto por duas etapas: inicialmente, análise de séries temporais^8^ das taxas, e, em seguida, avaliação de associações possíveis entre a variação percentual média das taxas e fatores contextuais.

### Fontes dos Dados

As informações sobre os casos são provenientes da base de dados do Sistema de Informação de Agravo de Notificação (SINAN), de acesso aberto^9^. A seleção das variáveis foi realizada de acordo com o período de notificação em cada UF e estratificadas por sexo. Foram utilizadas faixas etárias a partir de 13 anos e as categorias masculino e feminino da variável sexo. Para a caracterização da amostra, foram utilizadas as variáveis raça/cor autodeclarada, escolaridade e categoria de exposição hierarquizada.

Dados populacionais foram obtidos a partir de projeções e estimativas do censo demográfico do Instituto Brasileiro de Geografia e Estatística^10^. Variáveis contextuais foram obtidas a partir de dados do Programa das Nações Unidas para o Desenvolvimento^11^ (PNUD), do Instituto de Pesquisa Econômica Aplicada^12^ (IPEA) e do Ministério da Saúde^13^.

O Índice de Desenvolvimento Humano Municipal^11^ (IDH-M) possui medidas adimensionais entre 0 e 1, que classificam os valores de muito alto a muito baixo, com valores mais próximos de 1 indicando maior desenvolvimento humano. O Índice de Gini^14^ e o Índice de Vulnerabilidade Social^12^ (IVS) também assumem medidas entre 0 e 1, sendo os maiores valores indicativos de condições mais desfavoráveis. A proporção de distribuição de testes^13^ refere-se à razão entre quantidade de testes rápidos para diagnóstico de HIV distribuídos em cada UF e total de testes distribuídos no país. A taxa de analfabetismo^14^ é a proporção de indivíduos com 15 anos ou mais dentre todos da mesma faixa etária que declararam não saber ler ou escrever. A proporção de diagnóstico tardio^15^ se refere à proporção de indivíduos com resultados inferiores a 200 células/mL dentre todos os indivíduos infectados por HIV que realizaram o primeiro exame de linfócitos T CD4+ e que ainda não iniciaram o tratamento. Os indicadores contextuais utilizados neste estudo referem-se a cada uma das UF do Brasil. Foram obtidos os valores mais recentes disponíveis, considerando os anos do período do estudo.

### Análise Temporal das Taxas de Incidência de HIV/aids no Brasil

Nas análises das séries temporais, os anos do período de estudo (1 de janeiro de 2000 a 31 de dezembro de 2019) foram considerados variáveis independentes, e as taxas anuais de incidência de HIV/aids variáveis dependentes. Para o cálculo das taxas de incidência foram considerados o número de casos notificados de HIV/aids em cada UF, estratificados por sexo, e a população residente em cada UF no período, por sexo. As taxas de incidência foram padronizadas pelo método direto^16^. Foram realizadas, ainda, análises para a razão entre as taxas de incidência de ambos os sexos, considerando as razões entre as taxas para o estrato masculino em relação às taxas para o estrato feminino.

Modelos de regressão polinomiais foram utilizados, relacionando as taxas de incidência (Y) com o tempo em anos (X)


$$\text{Y}={\text{β}}_{0}+{\text{β}}_{1}\text{X}+{\text{β}}_{2}{\text{X}}^{2}+\in$$

em que a significância estatística de β_2_, que corresponde ao termo quadrático da regressão, indica melhor representação dos dados por relação não-linear. Para essas análises a variável tempo (em anos) foi centralizada a fim de evitar autocorrelação dos dados.

O modelo de regressão *joinpoint*^17^ foi empregado de forma a descrever a evolução temporal por pontos de inflexão que indicam modificação de tendências. Um modelo, dadas as observações, (x_1_, y_1_), …, (x_n_, y_n_), em que x_1_ ≤ … ≤ x_n_ e com τ_k_ pontos de junção desconhecidos, pode ser escrito por


$$\text{E}\left[\text{Y}|\text{X}\right]={\text{β}}_{0}+{\text{β}}_{1}\text{X}+{\text{δ}}_{1}{\left(\text{X}-{\text{τ}}_{1}\right)}^{+}\text{+}\cdots {\text{+δ}}_{\text{k}}{\left(\text{X}-{\text{τ}}_{\text{k}}\right)}^{+}$$

A inclusão de pontos de junção é testada pelo método de permutação de *Monte Carlo*, que utiliza uma sequência de testes a partir de uma amostra de permutações possíveis, com correção de *Bonferroni*^18^.

A partir deste método foram obtidas a variação percentual anual (APC, *annual percent change*) e a variação percentual média (AAPC) para o período completo. A APC corresponde a uma alteração linear da taxa percentual, estimada em escala logarítmica e considerando que x_1_, …, x_n_ representa os anos do período de estudo e y_1_, …, y_n_ representa o logaritmo das taxas observadas, sendo log (T_y_) o logaritmo da taxa no ano y. A variação percentual das taxas do ano y ao ano y + 1 corresponde a


$$\left[\frac{{\text{T}}_{\text{y}+1}+\text{Ty}}{\text{Ty}}\right]\times 100$$

Dessa forma, a APC é o percentual obtido pela exponencial da diferença entre as regressões de cada período e seu período antecedente. A AAPC, por sua vez, corresponde a uma medida resumida da tendência ao longo do intervalo de tempo fixado, e é calculada pela média ponderada das APC, com pesos iguais ao comprimento do intervalo.


$$\text{AAPC}=\left\{\exp\left(\frac{\sum {\text{w}}_{\text{i}}{\text{b}}_{\text{i}}}{\sum {\text{w}}_{\text{i}}}\right)-1\right\}\times 100$$

em que b_i_ se refere ao coeficiente de inclinação para o i-ésimo segmento, e w_i_ o comprimento de cada segmento no intervalo de anos.

### Associação da AAPC com Indicadores Contextuais

Na avaliação de possíveis associações entre a média da variação percentual das taxas de HIV/aids e os indicadores contextuais, foram consideradas variável dependente (Y) a média da variação do percentual anual (AAPC) de HIV e aids obtida na análise temporal, e variáveis independentes (X) os indicadores contextuais do estudo. Foram estimados modelos de regressão linear para análises univariadas e múltiplas.

Nas análises foi considerado nível de significância estatística de 5% e utilizado apoio computacional dos *softwares* R^19^, versão 4.1.3, e *Joinpoint Regression Program*^17^, versão 4.9.1.0.

### Considerações Éticas

Por utilizar dados secundários de aceso aberto, não foi necessária a submissão deste estudo para apreciação pelo Comitê de Ética em Pesquisa^20^.

## RESULTADOS

### Características Sociodemográficas e Epidemiológicas

Foram diagnosticados no Brasil 498.543 casos de HIV/aids em indivíduos do sexo masculino (64,01%) e 280.322 casos em indivíduos do sexo feminino (35,99%) com idade a partir de 13 anos entre 2000 e 2019. As taxas mais elevadas foram observadas para o sexo masculino: a menor taxa corresponde a 28,70/100.000, em 2006, e a maior 33,42/100.000, em 2000. Para o sexo feminino a menor taxa foi de 7,84/100.000, em 2000, e a maior 12,96/100.000, em 2009.

Na população com o agravo (Tabela 1) observa-se uma proporção importante de casos em jovens e adultos, com idade entre 20 e 49 anos (83,22% e 80,97% para os sexos masculino e feminino, respectivamente), e uma proporção maior em indivíduos com escolaridade fundamental incompleto, sendo mais expressiva para o sexo feminino (60,30%). Observa-se maior proporção de indivíduos pretos e pardos (46,20% para o sexo masculino e 30,41% para o sexo feminino). A transmissão por via sexual foi a mais frequente, predominantemente heterossexual para ambos os sexos, e mais expressiva para o sexo feminino (59,93%). A categoria “ignorado” apresentou proporções importantes para as variáveis raça/cor (37,55% para o sexo masculino e 40,43% para o sexo feminino) e categoria de exposição (42,57% para o sexo masculino e 36,72% para o sexo feminino).

**Tabela 1:** Características sociodemográficas dos casos de HIV/aids por sexo no Brasil no período de 2000 a 2019.

Características		Masculino		Feminino	
		n	%	n	%
Grupo etário					
	13–19	8.864	1,78	8.060	2,87
	20–29	120.643	24,2	66.575	23,75
	30–39	174.191	34,94	93.709	33,43
	40–49	120.076	24,08	66.701	23,79
	50–59	53.761	10,78	32.267	11,51
	≥ 60	21.008	4,21	13.010	4,64
Escolaridade					
	Analfabeto	7.901	2,9	6.318	4,84
	Fundamental incompleto	113.393	41,6	78.774	60,3
	Fundamental completo	23.583	8,65	14.377	11,01
	Médio incompleto	37.082	13,6	19.834	15,18
	Médio completo	46.115	16,92	1.919	1,47
	Superior incompleto	12.469	4,57	2.348	1,8
	Superior completo	32.056	11,76	7.056	5,4
Raça/cor					
	Branca	156.273	31,34	79.737	28,44
	Preta	32.386	6,5	21.574	7,7
	Amarela	1.773	0,35	929	0,33
	Parda	120.052	24,08	64.225	22,91
	Indígena	871	0,17	518	0,18
	Ignorado	187.188	37,55	113.339	40,43
Categoria de exposição hierarquizada					
	Homossexual	87.481	17,55	1.841	0,66
	Bissexual	29.718	5,96	811	0,29
	Heterossexual	144.145	28,91	167.997	59,93
	UDI	23.221	4,66	5.534	1,97
	Hemofílico	199	0,04	0	0
	Transfusão	130	0,03	106	0,04
	Acidente com material biológico	12	0,002	8	0,002
	Transmissão vertical	1.408	0,28	1.111	0,4
	Ignorado	212.229	42,57	102.914	36,72

UDI: usuários de drogas injetáveis.

### Evolução da Série Temporal

A análise de tendência por UF (Tabela 2) evidencia, para ambos os sexos, tendência estatisticamente significativa de crescimento (AAPC > 0) nas taxas de incidência para as UF das regiões Norte e Nordeste do país, ao passo que maiores tendências de declínio das taxas (AAPC < 0) são observadas entre as UF nas regiões Sul e Sudeste.

**Tabela 2:** Análise de tendência das taxas de HIV/aids por sexo nas unidades federativas do Brasil no período de 2000 a 2019.

UF	Sexo	Beta1	Beta2	R²	AAPC (%)
Acre (AC)	Masculino	0,51	-	0,58	**4,3**
	Feminino	0,02	-0,04	0,27	0,7
Alagoas (AL)	Masculino	0,99	0,03	0,92	**5,4**
	Feminino	0,36	-0,03	0,78	**4,6**
Amapá (AP)	Masculino	1,58	-	0,82	**5,9**
	Feminino	0,87	-0,06	0,73	**6,4**
Amazonas (AM)	Masculino	2,26	-0,15	0,85	**6,1**
	Feminino	0,55	-0,11	0,75	4,3
Bahia (BA)	Masculino	0,49	-0,03	0,9	**2,8**
	Feminino	0,15	-0,04	0,8	**1,7**
Ceará (CE)	Masculino	0,75	-0,02	0,93	**3,5**
	Feminino	0,08	-0,04	0,75	1,6
Distrito Federal (DF)	Masculino	-0,31	-0,09	0,29	-0,7
	Feminino	-0,65	-	0,75	**-4,2**
Espírito Santo (ES)	Masculino	0,23	-0,07	0,48	0,5
	Feminino	-0,49	-0,07	0,78	-2,2
Goiás (GO)	Masculino	0,37	-	0,68	1,6
	Feminino	-0,25	-0,02	0,74	-2,2
Maranhão (MA)	Masculino	1,39	-0,05	0,94	**6,3**
	Feminino	0,68	-0,07	0,93	**5,7**
Mato Grosso (MT)	Masculino	0,73	-	0,75	**2,6**
	Feminino	-0,11	-0,08	0,5	1,4
Mato Grosso do Sul (MS)	Masculino	0,84	-	0,7	**2,9**
	Feminino	0,09	-0,07	0,42	0,1
Minas Gerais (MG)	Masculino	-0,05	-0,02	0,25	-0,2
	Feminino	-0,39	-0,04	0,85	**-2,8**
Pará (PA)	Masculino	1,83	-	0,95	**8,5**
	Feminino	0,86	-0,06	0,93	**8,6**
Paraíba (PB)	Masculino	0,72	-	0,91	**4,1**
	Feminino	0,15	-0,04	0,73	**2,9**
Paraná (PR)	Masculino	-	-	0,05	0,3
	Feminino	-0,37	-0,05	0,67	-1,7
Pernambuco (PE)	Masculino	0,79	-0,05	0,91	**3,7**
	Feminino	0,21	-0,09	0,88	**2,4**
Piauí (PI)	Masculino	0,71	-0,05	0,8	**3,9**
	Feminino	0,27	-0,05	0,83	**4**
Rio de Janeiro (RJ)	Masculino	-0,61	-0,06	0,76	**-1,6**
	Feminino	-0,66	-0,08	0,87	**-2,1**
Rio Grande do Norte (RN)	Masculino	1,29	-	0,9	**7,3**
	Feminino	0,34	-	0,67	**4,5**
Rio Grande do Sul (RS)	Masculino	-0,51	-0,13	0,71	**-1,6**
	Feminino	-0,51	-0,2	0,86	-0,9
Rondônia (RO)	Masculino	1,02	-0,06	0,76	4
	Feminino	0,01	-0,11	0,82	-0,1

AAPC: do inglês *average annual percent change*.

Nota: valores em negrito indicam significância estatística a um nível de 5%.

As análises por razão entre as taxas de incidência por sexo indicam tendência quadrática, e a análise por pontos de inflexão indica tendência de queda na razão entre as taxas por sexo (AAPC = −1,8; IC95% −2,3 a −1,3). Observa-se redução na razão das taxas por sexo entre 2000 e 2008, seguida por leve aumento de 2009 a 2019. A razão das taxas de incidência entre os sexos apresenta tendência estatisticamente significativa de queda em 17 UF, indicando nestas localidades aumento nas proporções das taxas de incidência dos indivíduos do sexo feminino em relação ao sexo masculino.

No Brasil, as análises das regressões polinomiais (Figura 1) indicam tendência linear de declínio das taxas de incidência para o sexo masculino; a análise por pontos de inflexão apresentou tendência geral de redução nas taxas (AAPC = −0,6; IC95% −1,1 a 0,0). No modelo feminino observa-se tendência quadrática, com aumento das taxas de incidência no período de 2000 a 2009 e declínio entre 2010 e 2019. A análise por regressão segmentada apresenta tendência geral de crescimento nas taxas para o período completo do estudo (AAPC = 1,4; IC95% 0,8 a 1,9).

**Figura:** Tendência das taxas de HIV/AIDS (por 100.000) por sexo no Brasil no período de 2000 a 2019.

Nota: valores em negrito indicam significância estatística a um nível de 5%.

### Associação entre AAPC e Indicadores Contextuais

Dentre os modelos univariados (Tabela 3) que apresentaram significância estatística, o IDH-M apresenta os mais altos valores dos coeficientes para os sexos masculino e feminino (61,31 e 71,9, respectivamente). Os modelos univariados para taxa de analfabetismo, proporção de diagnóstico tardio e IVS apresentam associação direta com AAPC, ao passo que IDH-M e proporção de distribuição de testes apresentam associação inversa. O Índice de Gini não apresentou significância estatística.

**Tabela 3:** Resultados das análises univariadas de regressão da média da variação percentual anual (AAPC) das taxas de HIV/aids em pessoas do sexo masculino e feminino no Brasil no período de 2000 a 2019

Variáveis		Beta1	R²	p-valor
Masculino				
	Taxa de analfabetismo	0,39	0,39	**< 0,001**
	IDH-M	-61,31	0,69	**< 0,001**
	Índice de Gini	24,74	0,12	0,07
	Proporção de diagnóstico tardio	0,37	0,24	**< 0,01**
	Índice de Vulnerabilidade Social	29,06	0,29	**< 0,01**
	Proporção de distribuição de testes	-0,63	0,36	**< 0,001**
Feminino				
	Taxa de analfabetismo	0,46	0,4	**< 0,001**
	IDH-M	-71,9	0,71	**< 0,001**
	Índice de Gini	30,79	0,14	0,05
	Proporção de diagnóstico tardio	0,45	0,26	**< 0,01**
	Índice de Vulnerabilidade Social	33,76	0,29	**< 0,01**
	Proporção de distribuição de testes	-0,57	0,22	**0,01**

IDH-M: Índice de Desenvolvimento Humano Municipal.

Nota: valores em negrito indicam significância estatística a um nível de 5%.

O modelo múltiplo para o estrato masculino (Tabela 4) apresenta significância estatística para taxa de analfabetismo, IDH-M e proporção de distribuição de testes. Para o estrato feminino, apenas o IDH-M mantém significância estatística quando analisado conjuntamente com as demais variáveis independentes.

**Tabela 4:** Resultados da análise múltipla de regressão da média da variação percentual anual (AAPC) das taxas de HIV/aids em pessoas do sexo masculino e feminino no Brasil no período de 2000 a 2019

Variáveis (masculino)		Coeficientes	p-valor
Masculino				
	Taxa de analfabetismo	-0,26	**0,03**
	IDH-M	-64,98	**< 0,001**
	Índice de Gini	10,29	0,29
	Proporção de diagnóstico tardio	0,1	0,18
	Índice de Vulnerabilidade Social	3,67	0,69
	Proporção de distribuição de testes	-0,39	**< 0,001**
Feminino				
	Taxa de analfabetismo	-0,3	0,05
	IDH-M	-83,72	**< 0,001**
	Índice de Gini	15,67	0,22
	Proporção de diagnóstico tardio	0,13	0,19
	Índice de Vulnerabilidade Social	-0,48	0,97
Proporção de distribuição de testes		-0,25	0,06

IDH-M: Índice de Desenvolvimento Humano Municipal.

Nota: valores em negrito indicam significância estatística a um nível de 5%.

A análise dos coeficientes estimados para o modelo múltiplo do estrato masculino indica que, para cada ponto percentual de aumento na taxa de analfabetismo, o percentual anual médio das taxas de HIV/aids diminui em 26%. Para cada aumento no IDH-M (numa escala de 0 a 1), a AAPC diminui em aproximadamente 65%. Para cada ponto percentual de aumento na proporção de distribuição de testes, a média da variação percentual das taxas do agravo diminui em 39%. Referente ao sexo feminino, para cada aumento de 0 a 1 no IDH-M, a AAPC diminui em aproximadamente 83% quando condicionado aos demais preditores do modelo completo.

## DISCUSSÃO

As variações nas razões das taxas de incidência por sexo evidenciam que a proporção das taxas para os sexos masculino e feminino apresenta uma tendência geral de redução durante o período do estudo. Entretanto as tendências são distintas entre as UF do país. Aspectos como desigualdades sociais, vulnerabilidades e mudanças de tendências, assim como alcance do agravo em distintos segmentos populacionais, regiões e comunidades, já haviam sido apontados como associados a diferenças nas taxas entre os sexos e em diferentes localidades^3,4^. Estudos de tendência das taxas de acordo com os sexos, realizados em diferentes estados brasileiros, evidenciam variações nas razões estas variáveis, embora a proporção de casos entre indivíduos do sexo masculino seja sempre maior em relação ao sexo feminino^21^.

Estudos de tendência da epidemia identificaram mudanças nas taxas coincidindo com a implementação de políticas e práticas de saúde, o Tratamento como Prevenção (TcP) que visa interromper a cadeia de transmissão, a testagem no pré-natal, que tem se mostrado importante oportunidade de diagnóstico para mulheres em idade reprodutiva, e a implementação da notificação compulsória para casos de infecção por HIV sem diagnóstico de imunodeficiência. As tendências epidemiológicas se mostram consistentes com os benefícios das políticas de detecção precoce e acesso ao seguimento terapêutico pelos indivíduos diagnosticados^22,23^.

Neste estudo observou-se que as taxas de incidência de HIV/aids apresentam maiores magnitudes em estados das regiões Norte e Nordeste, onde também se observam as maiores tendências de aumento nas variações médias das taxas, ao passo que em estados nas regiões Sul e Sudeste as magnitudes das taxas observadas são menores e apresentam tendência de declínio. A epidemia, que no Brasil teve como polo inicial o estado de São Paulo, apresentou disseminação geográfica para estados nas regiões Norte e Nordeste desde a década de 1990, associada a fluxos migratórios em regiões fronteiriças. Esse padrão de dispersão, que envolve complexas dinâmicas de desigualdades sociais, contribuiu para os contornos da epidemia. As tendências das taxas nessas regiões se tornaram mais pronunciadas^24^.

A análise dos indicadores contextuais ressalta diferentes dimensões das desigualdades entre as diversas UF. Os resultados apontam para condições socioeconômicas caracterizadas por maior vulnerabilidade nas regiões Norte e Nordeste em comparação aos estados das regiões Sul e Sudeste. O IDH-M é o indicador que apresenta maior associação com a variação percentual anual de taxas, sendo estatisticamente significativa para ambos os estratos em todas as análises, e associação mais pronunciada para o sexo feminino. Vale especificar que os grandes valores dos coeficientes estimados para esse indicador no modelo múltiplo podem ser derivados da escala dos valores do IDH-M, que variam de 0 (mínimo) a 1 (máximo), portanto o aumento em uma unidade na variável IDH-M é equivalente a um aumento de 100% da escala, da pior condição para a melhor. Um estudo sobre tendências temporal e espacial e determinantes sociais de HIV/aids também encontrou associação positiva para a variável IDH-M^25^. No presente estudo, a proporção de diagnóstico tardio e o IVS apresentaram significância estatística nas análises univariadas, entretanto deixaram de apresentar significância quando analisados conjuntamente com os demais indicadores contextuais. Outro estudo evidenciou alta prevalência de diagnóstico tardio entre homens, apontando persistentes barreiras no acesso por indivíduos do sexo masculino aos cuidados em saúde que contemplam testagem para HIV^26^. Em outro estudo, o IVS foi utilizado nas análises de fatores associados à tuberculose, apresentando maior associação com as taxas em municípios caracterizados por maior vulnerabilidade^27^. Nos resultados aqui apresentados, a proporção de distribuição de testes e a taxa de analfabetismo revelaram significância estatística nas análises univariadas para ambos os estratos e mantiveram significância nas análises múltiplas apenas para o estrato masculino. Um estudo sobre a distribuição de testes aponta para aumento importante desde sua implementação e melhor provisionamento aos UF em todo o país^28^. Menores taxas de escolaridade são reconhecidas como fator que prejudica diversas condições da saúde e apresentaram associação com o maior número de diagnósticos de HIV/aids no Brasil^29^. Apesar de o Índice de Gini não apresentar associação com significância estatística nas análises aqui apresentadas, outro estudo evidenciou associação entre indicadores de melhores condições socioeconômicas e infecção pelo HIV, sugerindo desigualdade no acesso ao diagnóstico da infecção^30^.

Neste estudo observou-se predominância de casos e taxas de incidência com as maiores magnitudes em indivíduos do sexo masculino em relação a indivíduos do sexo feminino, e transmissão majoritariamente por via sexual. Esses achados são semelhantes aos dados oficiais do Ministério da Saúde^3^ e de estudos sobre perfis epidemiológicos realizados no Brasil^31^. Observa-se que ao longo do tempo a proporção de casos se mantém maior em indivíduos do sexo masculino, entretanto a via heterossexual passou a ser mais pronunciada, o que corrobora com os perfis de distribuição de casos aqui observados. Estudos recentes apontam para um aumento da incidência em faixas etárias mais jovens e com baixa escolaridade, o que pode indicar mudanças comportamentais relacionadas a práticas preventivas, diferenças no acesso ao diagnóstico, disponibilidade desigual dos serviços de saúde destinados a toda a população nacional, e o papel dos determinantes sociais raça e identidade étnica^32^. Consequentemente, a identificação de segmentos populacionais com maior vulnerabilidade e a realização de ações em saúde direcionadas podem promover igualdade social, equidade em saúde e melhorar o perfil da epidemia no Brasil.

Limitações do estudo incluem a utilização de base de dados quantitativos secundária, uma vez que não permite investigação qualitativa da pluralidade que caracteriza a vida das mulheres no país, como percepções de gênero, práticas sexuais e contextos de vulnerabilidades. Adicionalmente, variáveis como raça/cor e categoria de exposição apresentaram lacunas no preenchimento das fichas de notificação. Embora os estudos de séries temporais permitam vislumbrar o comportamento das taxas ao longo do tempo, as análises estão sujeitas a variações nos critérios diagnósticos e à disponibilidade de dados robustos e consistentes. Contudo, a possibilidade de situar as tendências aos contextos históricos e sociais permite uma melhor elucidação quanto aos pontos em que se observa modificações destas. Ressalta-se, ainda, que, por se tratar de estudo ecológico, não é possível fazer inferências em nível individual em relação aos resultados, no entanto essa restrição não compromete os achados, uma vez que os objetivos propostos foram direcionados ao nível populacional.

## CONCLUSÃO

A epidemia apresenta contornos diferentes de acordo com os sexos. Apesar de ser observado predomínio de casos em homens, a razão entre as taxas de incidência por sexos apresenta diferenças por região e período. Os casos de HIV/aids se distribuem de formas distintas na população feminina em relação à masculina, com tendência no Brasil de incremento nas taxas de incidência em mulheres e maior magnitude do efeito do indicador de desenvolvimento humano, o que pode apontar para uma associação entre o incremento nas taxas em mulheres e vulnerabilidades relacionadas ao gênero, sendo importante pensar em políticas públicas que considerem essas dimensões.
